# A Joint Global and Local Temporal Modeling for Human Pose Estimation with Event Cameras

**DOI:** 10.3390/s25092868

**Published:** 2025-05-01

**Authors:** Feifan Du, Zhanpeng Shao, Xueping Wang, Jianyu Yang, Jianhua Dai

**Affiliations:** 1 College of Information Science and Engineering, Hunan Normal University, 36 Lushan Road, Changsha 410081, China; duff2022@hunnu.edu.cn (F.D.); wang_xueping@hnu.edu.cn (X.W.); jhdai@hunnu.edu.cn (J.D.); 2 School of Rail Transportation, Soochow University, 8 Jixue Road, Suzhou 215100, China; jyyang@suda.edu.cn

**Keywords:** human pose estimation, event camera, joint global and local temporal modeling, attention mechanism

## Abstract

Event-based cameras, inspired by biological vision, asynchronously capture per-pixel brightness changes, producing streams of events with higher temporal resolution, dynamic range, and lower latency than conventional cameras. These advantages make event cameras promising for human pose estimation in challenging scenarios, such as motion blur and low-light conditions. However, human pose estimation with event cameras is still in its early research stages. Among major challenges is information loss from stationary parts of the human body, where the stationary parts at instances cannot trigger events. This issue, inherent to the nature of event data, cannot be resolved in a short-range event stream alone. Therefore, incorporating motion cues from a longer temporal range offers a intuitive solution. This paper proposes a joint global and local temporal modeling network (JGLTM), designed to extract essential cues from a longer temporal range to complement and refine local features for more accurate current pose prediction. Unlike existing methods that rely only on short-range temporal correspondence, this approach expands the temporal perception field to effectively provide crucial contexts for the lost information of stationary body parts at the current time instance. Extensive experiments on public datasets and the dataset proposed in this paper demonstrate the effectiveness and superiority of the proposed approach in event-based human pose estimation across diverse scenarios.

## 1. Introduction

In computer vision, human pose estimation aims to detect the positions of human keypoints from images or videos. As one of the challenging research areas, human pose estimation provides geometric and motion information about the human body for many downstream tasks, such as human–computer interaction, action recognition, and virtual reality. Therefore, it is essential to improve the accuracy and efficiency of human pose estimation. Although existing works have achieved significant performance [1,2,3,4], problems arise from challenging scenarios, such as motion blur and poor lighting conditions, for human pose estimation with conventional RGB cameras.

Unlike a conventional camera, which outputs dense representations at a fixed frame rate, an event camera asynchronously generates an event at a pixel only when the brightness change of that pixel exceeds a set threshold. An event consists of its location (*x*, *y*), timestamp (*t*), and polarity (*p*). Due to the imaging paradigm shift, event cameras possess competitive advantages over conventional cameras, including high temporal resolution, high dynamic range, low latency, and low power consumption. These properties make event cameras the best for addressing the above problems in challenging scenarios with conventional cameras [1,5,6,7,8]. However, meanwhile, a new issue arises from using event cameras; few events will be generated when the corresponding body part remain stationary in a time period, leading to body part information being missed.

**Figure 1:**
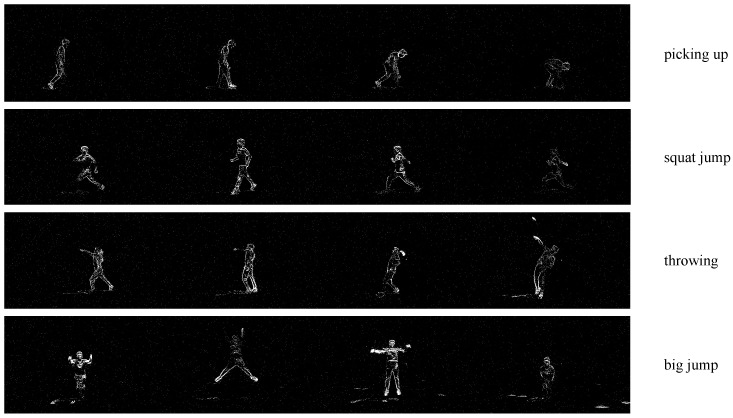
Event frames mapped from asynchronous event stream. Due to some body parts remaining stationary and not generating event information, certain body information is missing.

To address this issue, video-based methods [1,6,7,8] offer a potential solution by utilizing adjacent frames to provide complementary information for the current frame. However, for event data, missing body parts at a specific time instance may not always be recoverable from nearby frames within a short temporal range. These parts may only be identifiable in frames captured over a longer temporal span, as illustrated in Figure 1. Therefore, this paper proposes JGLTM to consider leveraging both the long-range temporal information distant from the current time instance and short-range adjacent information to offer motion cues for the human pose, while the motion consistency is kept for an event stream. The framework consists of three components. (1) For the purpose of long-range temporal modeling, JGLTM models a Global Memory Network (GMN) to iteratively abstract the long-range memory by applying a cross-attention block, where a learnable latent vector serves as the query input to continuously update the latent vector in the online recurrent manner. (2) JGLTM models the local spatial and temporal consistency of neighboring frames in a short range by building a local spatial–temporal transformer (LST), allowing the visual tokens of the current frame to interact not only with the tokens themselves but also with those of the adjacent frames. (3) Lastly, pose prediction is achieved by Mid-frame Prediction Refining (MPR) module, in which the global memory is used as the context to refine the feature of the middle frame in the LST module based on a cross-attention block.

With this joint global and local temporal modeling, JGLTM can take advantage of both short- and long-range information to enhance the feature at the current frame. Specifically, the feature is refined using a cross-attention mechanism to inject the global memory into the current frame, complementing the contextual motion cues and alleviating the issue of event loss. The contribution of this paper can be summarized as follows:

- This paper proposes a novel framework, JGLTM, for event-based human pose estimation. It explicitly captures both long-range contextual motion cues through the GMN module and short-range spatial–temporal consistency via the LST module, enhancing robustness to event sparsity. Furthermore, the MPR module is introduced, where global context from the GMN is injected into the middle frame’s features in LST using a cross-attention mechanism to improve prediction quality.
- This paper extends previous a dataset, CDEHP, to a larger benchmark CDEHP-E (CDEHP-E dataset is available at: https://cdehp-dataset.github.io/ accessed on 1 March 2025), including both indoor and outdoor scenarios, providing a more comprehensive evaluation platform.
- Extensive experiments on CDEHP, CDEHP-E, MMHPSD, and DHP19 demonstrate that the method proposed in this paper outperforms existing CNN-based and attention-based baselines, showing strong generalization across diverse conditions.

## 2. Related Work

### 2.1. Video-Based Human Pose Estimation

In the field of image-based human pose estimation, torso occlusion poses a significant challenge that necessitates considerable improvement. Similarly, the unique characteristics of event cameras, which generate event information only when brightness changes exceed a certain threshold, lead to accumulated grayscale images that suffer from information loss not only in the occluded torso regions but also in stationary body parts. Consequently, the primary challenge that must be addressed is how to retrieve the missing information from the neighboring frames of the target frame and effectively use it to enhance the target frame.

Early works [1,9,10] utilize additional optical flow information to supplement the data and impose constraints on the prediction results for the current frame. Specifically, Flowing ConvNets [9] aligns the heatmaps generated from neighboring frames with the current frame using optical flow estimation, resulting in warped heatmaps. They then fuse the information from the target frame’s heatmap with the warped heatmaps from neighboring frames through a 1 × 1 convolution kernel.

Similarly, Thin-Slicing Networks [10] extracts image features from neighboring frames using optical flow estimation to generate a structured model. This model effectively provides prior knowledge, regulates skeletal structures, and ensures spatiotemporal consistency, thereby reducing the likelihood of significant joint displacement. RPM [5] enhance geometric consistency between frames by utilizing LSTM memory units to store and update information from preceding and succeeding frames, building upon the CPM framework [11]. The authors of [12] propose a 3D extension of Mask R-CNN [13] to incorporate temporal information from neighboring frames into the current frame.

In general, adjacent frames should maintain consistency in both temporal and spatial dimensions. Addressing these aspects, ClipTrackingNetwork [14] transforms the problem of predicting the optimal position of each joint in every frame into a shortest path problem, solving it using Dijkstra’s algorithm. They generate clusters at hypothesized joint positions using the mean shift algorithm and enforce temporal consistency of clusters through a self-adaptive similarity function. DCPose [6] extracts temporal and spatial information through two parallel modules and inputs these data into a pose correction network, refining pose estimation with a narrowed search range and pose residual information. DiffPose [15] redefines the pose estimation task as a conditional heatmap generation task, using the visual features of the target and neighboring frames as conditions while applying a denoising diffusion probabilistic model for noise reduction. SLT-Pose [8] leverages self-attention to extract and refine features from both local sequence frames and the target frame. They input the refined features into a cross-attention module to establish associations between the target and neighboring frames, thereby supplementing the target frame with additional information. Meanwhile, FAMI-Pose [7] extracts additional relevant and complementary information from neighboring frames using feature alignment methods.

### 2.2. Event-Based Human Pose Estimation

Unlike dense data such as images, event information is sparsely distributed in space, making it challenging to use directly as input for neural networks. Consequently, various representations of event information have been proposed in prior works, raising the critical issue of selecting appropriate network structures for different representations. For event stream representation, the temporal information of events is preserved, making RNNs or Transformers suitable for capturing dynamic temporal features. For event point cloud representation, events are treated as spatiotemporal points, which can be effectively modeled using PointNet or GNNs to extract both local and global features. In this work, we adopt the event frame representation, where discrete events are accumulated into fixed-interval images, making CNNs a suitable choice for processing.

DHP19 [16] maps event information onto a 2D plane to generate event frames and employs a CNN to produce heatmaps. By setting a confidence threshold, they determine whether to update the joint positions in the current frame, thus mitigating information loss caused by stationary body parts. For each boundary pixel of the event frame, EventCap [17] searches for the closest event and solves a nonlinear least squares optimization problem to obtain boundary information, refining the pose accordingly. The authors of [18] input the sequence frames generated from the event stream into a CNN for optical flow estimation. They iteratively utilize the optical flow estimation and sequence frame information to generate pose estimation results. EventHPE [19] proposes a rasterized event point cloud that retains the 3D characteristics of event information, enhancing processing speed without compromising accuracy compared to directly projecting event information onto a 2D plane. MoveEnet [20] introduces an online, high-frequency, and lightweight network structure that uses the event stream directly as input. The event stream is converted into EROS representation and processed by a pre-trained network. Lastly, tDenseRNN [21] proposes a recurrent architecture featuring a novel temporal dense connection mechanism that integrates connections between the current frame and all preceding frames into a Long Short-Term Memory (LSTM) network, enabling comprehensive temporal modeling beyond simple sequential connections.

### 2.3. Vision Transformer for HPE

The application of transformer models, originally designed for translation tasks [22], to the traditional image domain Vit [23] has opened up a wider array of options beyond CNNs for various fields of computer vision, including human pose estimation. In Transpose [24], image features processed by a CNN are fed into the transformer’s encoder, uncovering image-specific and joint-specific dependencies through the derived dependency area.

In TokenPose [3], visual tokens are directly extracted from images, with new keypoint tokens introduced to learn features and predict joint positions, demonstrating a high similarity between keypoints and their neighboring or symmetric counterparts. The multi-resolution parallel design proposed in HRNet [2] is integrated into HRFormer [25], where self-attention is applied within non-overlapping small image windows to learn high-resolution representations. Additionally, 3 × 3 depthwise convolutions in two point-wise MLPs of the transformer facilitate information exchange between windows.

As the first transformer-based human pose estimation framework, TFPose [26] inherently reveals the structured dependencies among keypoints without the need for heuristic design. Building on TokenPose [3], PPT [4] introduces Human Token Identification (HTI) to locate a rough human body region, performing self-attention only within the selected tokens. In POT [27], a pose-oriented self-attention mechanism is proposed to explicitly model the topological interactions among body joints, while distance-related positional embeddings encode the distances from each joint to the root joint, effectively differentiating joint groups based on varying regression difficulties. Leveraging the sparse nature of event information, EventTransformer [28] presents a patch-based event data representation to reduce computational resource requirements. This approach introduces latent memory vectors to learn features and generate heatmaps, updating the latent memory vectors with each new frame when multiple frames are processed. In [29], a transformer is utilized to provide global spatial information, dynamically adjusting the spiking threshold of the SNN module.

## 3. Method

**Figure 2:**
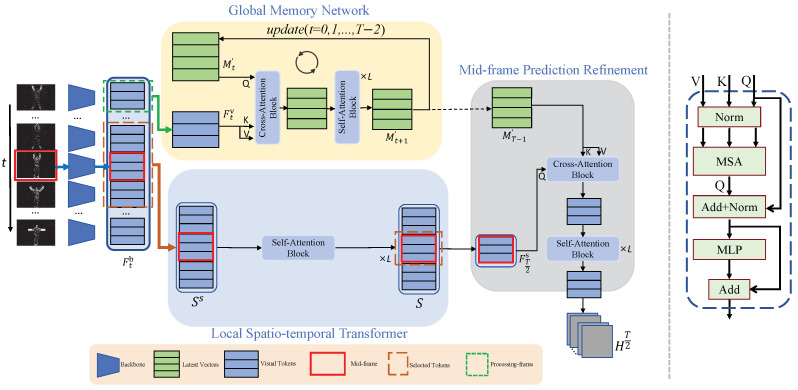
(**left**) An overview of network proposed in this paper. Event frames are fed into backbone to obtain visual tokens, which are then processed by the GMN module (highlighted in the orange background) to extract information from the processing frame. After processing all input frames, GMN acquires global pose information *M*. Concurrently, LST inputs the visual tokens from the middle frame and its neighboring frames into Self-Attention Block to facilitate the exchange of spatio-temporal information. Next, MPR combines the visual tokens from the middle frame with *M* and inputs them into Cross-Attention Module to complete the information for the middle frame and generate the final heatmap. (**right**) Architecture shared by the Cross and Sel-Attention Blocks.

In this section, the JGLTM method, consisting of three major components, is introduced, as shown in Figure 2(left). The detailed approaches are described in the following subsections, including feature extraction, local spatio-temporal transformer, global memory network, and finally, mid-frame prediction refining, which considers taking advantage of long-short temporal abstract memory to refine the local feature.

### 3.1. Preliminary Details

In this paper, a self-attention block is used to further extract input features and a cross−attention block to acquire global memory, continuously extracting global information from the input features. The self-attention block and cross-attention block have the same structure, except for the input, as shown in Figure 2(right). This structure mainly consists of MSA, MLP, Norm, and Add operations. Next, a detailed explanation of MSA and MLP is provided.

#### 3.1.1. Multihead Self-Attention(MSA)

The formula for the self-attention mechanism is expressed as in Equation (1):

(1) $$Attention\left(Q,K,V\right)=softmax\left(\frac{QK^{T}}{\sqrt{d_{k}}}\right)\times V,$$

where *Q* is the query matrix, *K* is the key matrix, *V* denotes value matrix, and $d_{k}$ represent the dimension of *Q* and *K*.

The MSA module is a combination of several self-attention mechanism modules, given by Equation (2):

(2) $$\begin{array}{cc} MSA(Q,K,V) & =Concat\left(head_{1},\cdots ,head_{J}\right)W^{O}\\ head_{j} & =Attention\left(QW_{j}^{Q},KW_{j}^{K},VW_{j}^{V}\right), \end{array}$$

where $W^{O}$, $W_{j}^{Q}$, $W_{j}^{K}$ and $W_{j}^{V}$ are the projection parameter matrices. Projection refers to using a linear transformation to map a vector from one space to another. In neural networks, it is typically implemented through matrix multiplication to adjust the dimensionality and feature representation of the input data.

#### 3.1.2. Multilayer Perceptron(MLP)

The MLP is mainly composed of two fully connected layers and a linear activation layer.

(3) $$MLP=max\left(0,XW_{1}+b_{1}\right)W_{2}+b_{2},$$

where $W_{1}$, $b_{1}$, and $W_{2}$, $b_{2}$ represent the weights and biases of the two fully connected layers, respectively.

In addition, a Layer Normalization (LN) layer is applied before each module, and a skip connection is employed after each module. Thus, each transformer layer can be expressed as in Equation (4):

(4) $$\begin{array}{cc} Q_{out} & =MSA(LN(Q),LN(K),LN(V))+Q\\ Self/Cross & -AttenBlock\left(Q,K,V\right)=MLP\left(LN\left(Q_{out}\right)\right)+Q_{out}. \end{array}$$

### 3.2. Feature Extraction

With the variation of light intensity in the environment, an event camera outputs an event e={x,y,ts,p}, where (x,y) represents the pixel location where the event is triggered, ts denotes the timestamp, and *p* indicates the trend of intensity change 1 for increase, −1 for decrease). In this paper, each event stream is segmented along the temporal dimension into a sequence of event packets $\epsilon =\{e_{1},e_{2},e_{3},\cdots \}$, where each event $e_{m}$ occurs within a time interval Δt. Each event packet is then accumulated onto a 2D plane using the method in [21] to generate a 2D grayscale frame $I\in R^{H\times W}$. JGLTM adopts a CNN backbone HRNet-w32 pretrained on the ImageNet to extract frame features.

(5) $$F_{t}^{a}=HRNet-w32\left(I_{t}\right),t=0,1,2,\cdots ,T-1.$$

Here, *T* represents the number of event frames input into the network. After the feature extraction, JGLTM divides the feature map $F_{t}^{a}\in R^{H_{a}\times W_{a}\times C}$ into patches and flattens it into 1D vectors to obtain visual tokens $F_{t}^{b}\in R^{N\times (P^{2}\times C)}$, where ($H_{a}$, $W_{a}$, *C*) is the shape of the feature map, (*P*, *P*, *C*) is the shape of each patch, and *N* = $H_{a}W_{a}$/$P^{2}$ is the number of patches. To perform cross-attention computation with the latent vectors, we feed the extracted visual tokens through an MLP to align the dimension with the latent vectors $M_{t}^{\prime }\in R^{B\times D}$, and *B* is the number of latent vectors, $F_{t}^{v}=MLP\left(F_{t}^{b}\right)$. Here, $F_{t}^{v}\in R^{N\times D}$ is used to represent the processed visual tokens.

### 3.3. Local Spatio-Temporal Transformer

Alongside extracting the global feature matrix *M*, JGLTM also obtains the visual tokens from all image frames. Since nearby frames significantly influence the prediction of the current frame, it is crucial to extract both spatiotemporal information from neighboring frames and spatial information from the current frame. To achieve this, JGLTM inputs the visual tokens of the target frame along with its neighboring frames, denoted as $S^{s}={\left\{F_{t}^{v}\right\}}_{t=\frac{T}{2}-w}^{\frac{T}{2}+w}\left(S^{s}\in R^{((2w+1)\times N)\times D}\right)$, into self-attention blocks. This allows for the exchange of spatio-temporal information, capturing local details more effectively.

(6) $$S=Self-AttenBolck(S^{s},S^{s},S^{s}).$$

Subsequently, JGLTM extracts the visual tokens of the mid-frame, where each token has exchanged information with both the visual tokens of nearby frames and its own internal tokens. The extracted tokens, denoted as $F_{\frac{T}{2}}^{s}\in R^{N\times D}$, are obtained from the output of the self-attention blocks.

### 3.4. Global Memory Network

Similar to [28], JGTLM initializes latent vectors as memory to extract and store event frame information. By continuously processing the features of the current input frame, the memory is gradually refined and updated, resulting in global information encompassing all key details from the input frames. Next, the process of refining and updating the memory will be explained in detail.

#### 3.4.1. Memory Refinement

To further refine the memory based on previous frames, JGLTM inputs the current frame’s information along with the latent vectors into a Cross Attention Module, which consists of a cross-attention Block and K self-attention blocks. In this setup, the visual tokens serve as keys (K) and values (V), while the latent vectors act as queries (Q). Each visual token is added with position embedding to preserve its relative position in the original frame.

#### 3.4.2. Memory Update

To supplement and enhance the memory, JGLTM adds the refined memory to the previous memory using a simple sum operation. This updated memory $M_{t}^{\prime }$ is then used to extract features for the next frame in subsequent processing. After all frame inputs are completed, the latent vectors are updated to obtain the final $M\in R^{B\times D}$.

(7) $$\begin{array}{c} M_{t+1}^{\prime }=CrossAttenModule\left(M_{t}^{\prime },F_{t}^{v},F_{t}^{v}\right)+M_{t}^{\prime }\\ M=M_{T-1}^{\prime }. \end{array}$$

### 3.5. Mid-Frame Prediction Refinement

In order to supplement the global information of the current frame and mitigate the issues of local stillness in human poses and torso occlusion, JGLTM injects the latent vectors representing global information, obtained from the previously introduced GMN module, into the refined middle frame information produced by the LST module. JGLTM inputs the processed visual tokens along with the final result *M* of GMN into a Cross Attention Module which is the same as in the previous GMN module. Here, the visual tokens $F_{\frac{T}{2}}^{s}$ are treated as Q, *M* as K and V, in contrast to the previous step of extracting the image frames.

(8) $$F=CrossAttenModule(F_{\frac{T}{2}}^{s},M,M).$$

Finally, in order to better predict the pose, JGLTM concatenates the rough information $F_{\frac{T}{2}}^{a}\in R^{h\times w\times \left(\frac{H_{a}\times W_{a}\times C}{h\times w}\right)}$ extracted by the previous backbone with the current refined information $F\in R^{h\times w\times (\frac{N\times D}{h\times w})}$ to obtain more comprehensive feature information. Next, it is passed through a 1×1 convolution kernel to adjust the dimensions of the feature map corresponding with the number of keypoints, resulting in the final heatmap $H^{\frac{T}{2}}\in R^{h\times w\times numJoints}$, where *h* and *w* represent the size of the heatmap.

(9) $$H^{\frac{T}{2}}=ConvKernel_{1\times 1}\left(F⨁F_{\frac{T}{2}}^{a}\right).$$

### 3.6. Training of the Network

For each event frame input into the network, we only output a mid frame heatmap for prediction. We use the MSE loss function to compare the predicted heatmaps with the ground truth heatmaps.

(10) $$Loss=\sum_{k}^{K}∥H_{\frac{T}{2}}\left(k\right)-H_{\frac{T}{2}}^{*}\left(k\right)∥,$$

where $H_{\frac{T}{2}}\left(k\right)$ and $H_{\frac{T}{2}}^{*}\left(k\right)$ are the predicted heatmaps and ground truth for the k-th joint in the mid event frame, respectively.

### 3.7. CDEHP-E Dataset

CDEHP is a multimodal human pose dataset captured in outdoor scenes and currently stands as the most challenging event camera-based human pose dataset. To better leverage the high dynamic range characteristics of event cameras, we followed the data collection and annotation methods used in the CDEHP dataset to create an indoor dataset. This indoor dataset comprises samples from 10 participants, as shown in Table 1, each performing 13 different actions at varying speeds (slow, medium, and fast) for 3 to 4 repetitions over a period of time. In total, we collected 130 video samples, each consisting of RGB video sequences, depth video sequences, and event streams. This results in approximately 45,000 frames of RGB-D data. We then combined the collected indoor dataset with the outdoor dataset from CDEHP, resulting in a new dataset named CDEHP-E, as shown in Table 2.

## 4. Experiment

### 4.1. Experiment Setup

#### 4.1.1. Dataset

We evaluate method proposed in this paper on four datasets: DHP19 [16], MMHPSD [19], CDEHP [21], and CDEHP-E. The DHP19 dataset comprises 33 movement recordings from 17 subjects (12 females and 5 males), aged between 20 and 29. The movements are categorized into upper-limb, lower-limb, and whole-body movements, distributed across 5 sessions. MMHPSD is the largest event-based 3D human pose and shape dataset, featuring recordings of 15 subjects (11 males and 4 females). Each subject performs 3 groups of actions (21 distinct actions in total) four times, with each group including actions executed at fast, medium, and slow speeds. This results in 180 videos, each approximately 1.5 min long, and a total of 240,000 grayscale images. The CDEHP dataset is a multi-modal human pose dataset captured in outdoor settings, including samples from 20 subjects (15 males and 5 females) recorded in four different outdoor environments. Each subject performs 25 distinct actions at varying speeds (slow, medium, and fast) 3 to 4 times, resulting in a total of 101,000 frames and 300 event streams in the dataset.

#### 4.1.2. Implementation

The proposed method and comparison approaches in this paper all use event frames as network inputs. Since DHP19 contains only event data, we accumulate the events in each event stream to generate event frames. To focus on the pose estimation with event cameras, we assume the detection stage has been completed with human areas in frames detected, and we crop event frames to a fixed size of 256 × 256 with the human bodies set at the center. The backbone we used in our network is HRNet-w32, which is a specific variant of HRNet [25]. The number of self-attention blocks in the Cross-Attention module *L* is set to 3. The temporal length of input frames T is set to 9. The temporal length of nearby frames *w* is set to 1. The number of latent vectors (*B*) and the number of visual tokens (*N*) are set to 64. The size of the patch generated by cutting the feature map P is set to 8. Dimension of latent vectors and visual tokens (*D*) are set to 512. The Adam optimizer is initialized with a learning rate of 1e-4 and adjusted using a cosine annealing schedule with a cycle of 32. Model convergence is determined based on the stability or slight fluctuations of the AP metric on the validation set. According to experimental observations, the total number of training epochs is set to 32 for the DHP19 and MMHPSD datasets, and 64 for the CDEHP and CDEHP-E datasets, when performance tends to plateau. The training batch size is set to 32, and data augmentation includes rotation, random scaling, and random flipping. The model is trained on four NVIDIA 2080Ti GPUs.

#### 4.1.3. Evaluation Metric

We utilize average precision (AP) and percentage of correct keypoints (PCK) as our evaluation metrics on the CDEHP and MMHPSD datasets. The AP metric is computed based on Object Keypoints Similarity (OKS) [30], which quantifies the similarity between two sets of keypoints. Specifically, we report $AP^{50}$ (AP at OKS = 0.50), $AP^{75}$ (AP at OKS = 0.75), and AP (the mean AP over OKS thresholds from 0.50 to 0.95, with a step of 0.05). For the PCK metric, a detected joint is considered correct if the Euclidean distance between the predicted and ground-truth locations falls within a certain threshold. In our evaluation, we use PCK@0.5, where a joint is deemed correctly detected if it lies within 0.5 × head_bone_length of the ground truth. Since AP and PCK tend to saturate on DHP19 when using a simple network, making it unsuitable for verifying our model’s performance, we additionally employ the mean per joint position error (MPJPE) as an evaluation metric for the DHP19 dataset. MPJPE measures the mean L2 distance between the predicted and ground-truth keypoints, defined as $\frac{1}{K}\sum_{k=1}^{K}∥p_{k}-p_{k}^{*}∥$. Here, $p_{k}$ and $p_{k}^{*}$ represent the ground-truth and predicted positions of the *k*-th joint in the image space, respectively, providing a more accurate evaluation of keypoint localization performance.

### 4.2. Experimental Analysis

#### 4.2.1. The Influence of Input Resolution

To analyze the impact of input event frame resolution on model accuracy, we conducted experiments at resolutions of 224 × 224, 256 × 256, and 384 × 384. To eliminate the influence of the varying numbers of visual tokens, we set the patch sizes to 7 × 7, 8 × 8, and 12 × 12, respectively. This ensures that the model segments the feature maps generated by the convolutional network into 64 visual tokens and uses 64 as the input for latent vectors.

As shown in Table 3, the AP does not consistently increase with higher image resolutions. When the resolution increases from 224 × 224 to 256 × 256, the AP improves by 1.62, indicating that larger patches provide richer feature information, thereby enhancing prediction accuracy. However, further increasing the resolution to 384 × 384 leads to a drop in AP by 2.37. Although higher-resolution patches contain denser information, they may also introduce more background noise or redundant details, which can negatively affect the model’s discriminative ability. Additionally, the model may struggle to effectively utilize the added fine-grained information. Overall, 256 × 256 achieves a good balance between informative content and irrelevant noise, making it the optimal patch granularity for the current task.

#### 4.2.2. Effect of the Global Temporal Length

**Figure 3:**
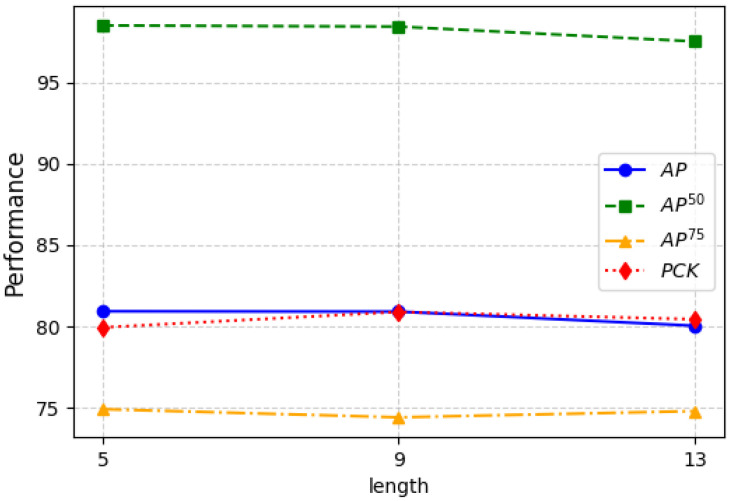
Ablation studies of different temporal lengths of input frames on CDEHP dataset.

Global temporal length refers to the length of the event frame sequence we input into network. By extracting features from the entire input sequence and updating the latent vectors, we ultimately obtain global pose information. A short event frame length may result in insufficient temporal information, making it difficult for the model to capture motion patterns, while an excessively long frame length may introduce redundant information, reducing computational efficiency. Therefore, this paper selects multiple frame lengths (5, 9, and 13) to cover short, medium, and long time spans of event information, enabling an analysis of the impact of time window size on model performance. As shown in Figure 3, although the AP metric slightly decreases when the length is 9, there is a significant improvement in PCK. When the length increases to 13, both AP and PCK decrease to varying degrees. The experimental results indicate that a longer global temporal length for generating global pose information is not necessarily better, and an overly long sequence input can interfere with the generation of global pose information. Therefore, even though the AP metric is not optimal at a length of 9, considering all factors, we set the global temporal length to 9 in our experiments for this paper.

#### 4.2.3. Effect of the Local Temporal Length

**Figure 4:**
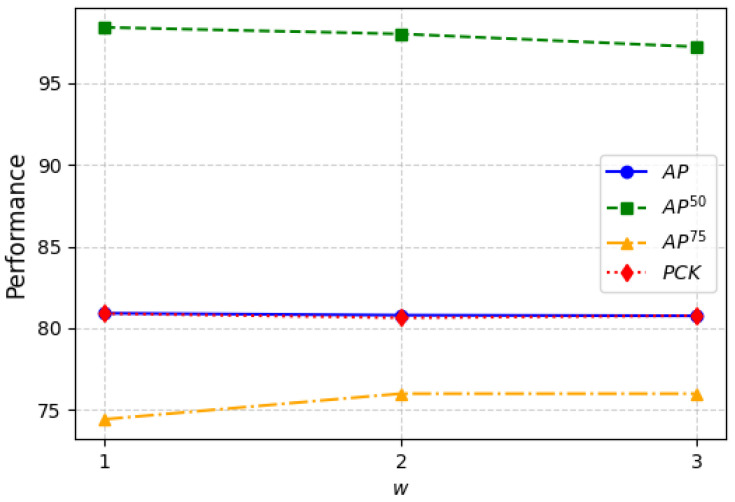
Ablation studies of different lengths (*w*) of nearby frames on CDEHP dataset. Here, w=2 means the total frames fed to the LST module will be 2+2+1=5, including the mid-frame.

The nearby temporal length is a hyperparameter of our model, representing the number of frames taken from the prediction of nearby frames. As shown in the results in Figure 4, with the increase in *w*, most evaluation metrics such as AP, $AP^{50}$, and PCK tend to decrease, while $AP^{75}$ shows a slight improvement. This indicates that as the number of frames for local information exchange increases, the overall prediction accuracy of our model slightly decreases, but OKS improves to above 75% for some inputs. The results suggest that more local information is not always better and excessive information exchange can lead to the prediction frame deviating from the correct trajectory. This indicates that more temporal information does not always lead to better predictions. An excessively long time window may introduce redundant information, cause prediction drift, and increase computational complexity. Therefore, choosing an appropriate *w* helps balance capturing local temporal information and maintaining prediction accuracy. Considering these findings, we set *w* to 1 for the experiments in this paper.

Additionally, we found that PCK and AP exhibit very similar performance. PCK evaluates whether keypoint predictions fall within a certain error threshold, while AP measures the precision and recall of keypoint detection. This similarity may be attributed to the stable confidence scores of JGLTM on the CDEHP dataset and the relatively uniform error distribution, leading to comparable trends in AP and PCK results.

#### 4.2.4. Contribution of Each Component

Our model is primarily composed of three parts: extraction of global or local information, and infilling local prediction frames with global information. To validate the effectiveness of our modules, we conducted extensive experiments. First, we removed the GMN module from our proposed network and replaced the *K* and *V* inputs of MPR with *Q* information to avoid global information filling. We denote this configuration as JGLTM-w/o-global. Next, we set the number of neighboring frames in the LST module to 0 to prevent the middle frame from obtaining information from adjacent frames, referred to as JGLTM-w/o-local.

The results in Table 4 clearly show that removing either global or local information significantly impacts the model’s performance. Compared to JGLTM, the model without global information shows a 0.65 decrease in AP and a 1.71 decrease in PCK, while the model without local information exhibits a 1.11 decrease in AP and a 1.5 decrease in PCK. Additionally, we observe that removing global information has a more substantial effect on PCK, whereas removing local information impacts AP more significantly.

### 4.3. Comparisons with State of the Art

#### 4.3.1. Results on DHP19

**Figure 5:**
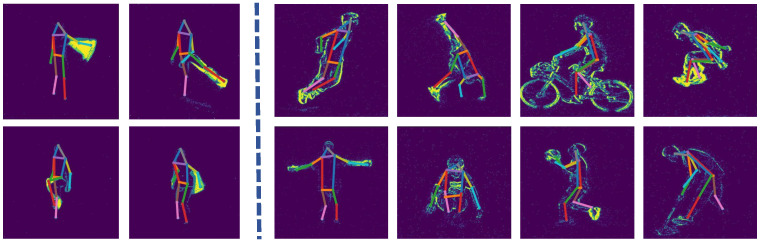
Visual results of our JGLTM on DHP19 (**left**) and CDEHP-E (**right**). Challenging scenarios including fast motion and mutual occlusion are involved.

Table 5 summarizes the results of various methods on the DHP19 dataset. Our proposed method shows a decrease of 0.17 MPJPE and 0.43 MPJPE compared to tDenseRNN [21]. Furthermore, we observe that the differences between other methods and our proposed method are minimal. This is likely due to the relatively simple pose information collected in the DHP19 dataset, which does not effectively evaluate the performance of our method. Partial prediction results of DHP19 can be viewed in Figure 5.

#### 4.3.2. Results on CDEHP

In Table 5, we present a comparison of our model with other state-of-the-art models based on the evaluation metrics AP and PCK. Since GNN networks with point-based representations still show a significant performance gap compared to event frame-based methods, we do not include them for comparison in this paper. Among the compared models, Hourglass [31], SimpleBaseline [1], HigherHRNet [30], TokenPose [3] and VitPose [33] are representative algorithms for human pose estimation based on images, achieving AP scores of 75.87, 77.51, 75.60, 79.68 and 80.01, respectively. LSTM-CPM [5], DKD [32], DCPose [6], FAMI-Pose [7] and tDenseRNN [21] are representative algorithms based on videos, achieving AP scores of 59.37, 78.97, 77.56, 79.33 and 80.18, respectively. Compared to tDenseRNN, which achieved the best results on videos, our model shows an improvement of 1.29PCK and 0.73AP, respectively. The above comparison demonstrates that our model achieves excellent performance in event-based human pose estimation. The modules within our model effectively extract both global and local pose information, and the infusion of global information into local details optimizes pose estimation.

#### 4.3.3. Result on CDEHP-E

We observe that all methods show significant improvements compared to their results on the CDEHP-E dataset. Our proposed JGLTM achieves better performance on the CDEHP-E dataset, with improvements of 2.81AP and 3.15PCK compared to the results from CDEHP. To investigate the reason behind the observed performance improvement, we conducted experiments based on the CDEHP training set, as shown in Table 6. We added additional indoor data to the training set and evaluated the results on the CDEHP validation set. Compared to the original training set without indoor data, the AP and PCK dropped by 1.28 and 1.86, respectively, suggesting that adding indoor data did not benefit training for outdoor validation. This asymmetry may be attributed to the fact that indoor data tend to be less noisy and more structured than outdoor data, making it easier for the model to learn and make accurate predictions. Partial prediction results of CDEHP-E can be viewed in Figure 5.

#### 4.3.4. Results on MMHPSD

We achieved similar results on the MMHPSD dataset as we did on the CDEHP dataset. Compared to tDenseRNN, our model shows an improvement of 3.08 in AP and 1.94 in PCK. The greater improvement on the MMHPSD dataset compared to the CDEHP dataset suggests that our model performs consistently well across datasets of varying complexity.

#### 4.3.5. Action-Wise Result Comparison on CDEHP Dataset

To analyze the performance of our method on different actions, we report the action-wise results on the CDEHP dataset. As shown in Table 7, the actions in the CDEHP dataset are categorized into slow, medium, and fast movements. Our method achieves the best results for the majority of actions, particularly excelling in slow and medium movements. This indicates that our approach effectively utilizes global pose information to fill in missing body part data caused by stationary positions during slow movements. The results of FAMI-Pose and tDenseRNN are relatively close to our method. To our understanding, tDenseRNN achieves results that are close to our method in the action-wise evaluation by utilizing dense connections and attention maps and FAMI-Pose achieves this performance through leveraging feature alignment methods to extract additional relevant and complementary information from neighboring frames. Among the other methods we compared, Hourglass performs best on jumping jacks, SimpleBaseline excels in spinning and jumps in various directions, while TokenPose achieves the best results in big jumps and crotch high five. Furthermore, we observe that in actions like crawling, cartwheeling, spinning, and long jumps, severe body part occlusion prevents all methods from achieving satisfactory performance.

### 4.4. Result Visualization

**Figure 6:**
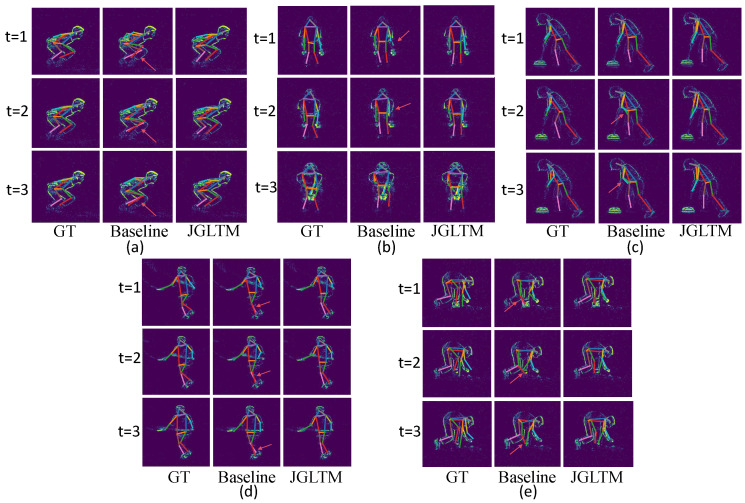
Visual results on the CDEHP dataset using our model JGLTM and the baseline model JGLTM+w/o global, respectively. (**a**–**e**) are visualization of five differentiated actions: (**a**) frog jumping, (**b**) crotch high five, (**c**) sweeping, (**d**) throwing, and (**e**) picking up.

In Figure 6, we visualize some of the prediction results on our model and JGLTM-w/o-global from the test set. These include frontal (b,d) and side poses (a,c,e), as well as cases with body part occlusion (a,d,e) and partial body information loss (b,c). We observe that in situations where body parts are occluded, the baseline’s predictions often show significant deviations for certain joints (e.g., the left foot and right knee in (a), both feet in (d), and the right knee and right foot in (e)), leading to partial distortion of the pose. When body parts remain stationary and generate less event information, the baseline predictions show inconsistencies over time (e.g., the left hand in (b) is correctly predicted at t=3 but incorrectly at t=1 and t=2; the hands in (c) are correctly predicted at t=1 but are incorrect at t=2 and t=3).

## 5. Conclusions

This paper presents JGLTM for human pose estimation based on event information. JGLTM introduces a local attention mechanism to facilitate information exchange between the middle frame and nearby frames, thereby supplementing information for the target frame. Furthermore, to address the challenges of event cameras in capturing event information with stationary body parts and occlusions, JGLTM incorporates a global memory network to extract global pose information and use it to fill in the middle frame, enhancing pose prediction accuracy. Additionally, to demonstrate the universality of our method, this paper collects a substantial amount of indoor human pose data to supplement the CDEHP dataset. Experiments conducted on four datasets validate the effectiveness of our method for event-based human pose estimation, achieving the best results. Although frame-based event representation has demonstrated strong performance in human pose estimation, it has not fully taken advantage of the asynchronous and low-latency properties of event cameras, while the frame-based representation requires extra accumulation preprocessing. Moreover, this representation overlooks the inherent sparsity of event data and does not fully leverage its temporal information. Future research can focus on enhancing computational efficiency and exploiting the unique properties of event data to facilitate the deployment of event cameras in low-power, real-time computing scenarios. 

## Figures and Tables

**Table 1:** Lists of recorded human actions performed at low, medium, and fast speeds.

Speed	Action
slow	walking, picking up, sweeping
medium	squat jumping, boxing, suping jumping,
	kicking, spinning, throwing
fast	open and close jumping, crotch high five,
	alternating squat jump, spreading arm big jump

**Table 2:** Existing event-based human pose datasets are compared in terms of the number of subjects (Sub#), the number of actions per subject (Act#), the number of frames (Frame#), the total duration, the resolution of event camera, the number of used event cameras and multi-modality (MM). The shooting scenes are also listed for comparison.

Dataset	Sub#	Act#	MM	Frame#	Total Duration (min)	Resolution	Event Camera Number	Scenes
DHP19	17	33	No	87 K	196.35	346 × 260	4	indoor
MMHPSD	15	12	Yes	240 K	270	1280 × 800	1	indoor
CDEHP	20	25	Yes	101 K	14.09	1280 × 800	1	outdoor
CDEHP-E	30	25	Yes	146 K	20.32	1280 × 800	1	outdoor & indoor

**Table 3:** The performance of JGLTM with different resolutions on CDEHP val set.

Resolution	Patch Size	*N*	*B*	AP
224 × 224	7 × 7	64	64	79.29
256 × 256	8 × 8	64	64	80.91
384 × 384	12 × 12	64	64	78.54

**Table 4:** Ablation studies on contributions of different components on CDEHP dataset. ↑ indicates better performance with higher values. The best results are highlighted in bold.

Method	*AP ↑*	*${\mathrm{AP}}^{50}$ ↑*	*${\mathrm{AP}}^{75}$ ↑*	*PCK ↑*
JGLTM-w/o-local	79.80	97.64	70.47	79.39
JGLTM-w/o-global	80.26	97.38	72.66	79.18
JGLTM	**80.91**	**98.43**	**74.41**	**80.89**

**Table 5:** Comparison with state-of-the-art methods on the CDEHP, MMHPSD, DHP19 and CDEHP-E datasets. ↑ indicates better performance with higher values, ↓ signifies better performance with lower values. The best results are highlighted in bold, second-best underlined. The input size for all methods is 256 × 256.

Method	CDEHP [21]				MMHPSD [19]				DHP19 [16]	CDEHP-E			
	AP ↑	${\mathrm{AP}}^{50}↑$	${\mathrm{AP}}^{75}↑$	PCK↑	AP↑	${\mathrm{AP}}^{50}↑$	${\mathrm{AP}}^{75}↑$	PCK↑	MPJPE↓	AP↑	${\mathrm{AP}}^{50}↑$	${\mathrm{AP}}^{75}↑$	PCK↑
Hourglass [31]	75.87	91.78	59.47	71.32	76.47	94.88	65.55	91.74	7.18	79.12	96.15	68.83	74.87
SimpleBaseline [1]	77.51	93.10	63.20	73.60	77.16	95.12	67.73	91.84	7.15	80.03	96.76	71.05	75.98
HigherHRNet [30]	75.60	91.65	57.95	71.56	78.18	95.53	70.62	92.14	7.02	78.92	96.36	67.71	73.94
LSTM-CPM [5]	59.37	67.63	28.10	49.07	40.99	39.28	3.66	54.75	7.36	62.59	70.33	43.65	53.66
DKD [32]	78.97	95.37	67.36	76.79	81.07	97.44	77.90	94.41	5.40	81.45	97.52	75.63	78.95
DCPose [6]	77.56	93.65	63.18	74.80	81.97	97.45	80.62	95.02	6.62	80.42	96.96	73.48	76.84
TokenPose [3]	79.69	97.49	70.61	75.80	85.58	98.56	89.44	97.16	5.24	82.45	97.98	77.73	79.73
FAMI-Pose [7]	79.33	97.75	68.29	80.19	85.22	98.61	89.11	98.83	5.44	82.31	**98.88**	80.15	82.23
VitPose [33]	80.01	97.67	71.20	74.79	82.53	99.05	86.10	94.79	5.83	82.16	98.03	80.68	77.98
tDenseRNN [21]	80.18	95.51	71.50	79.70	86.96	99.09	91.77	97.14	5.08	82.76	96.35	78.05	82.31
JGLTM-w/o-global	80.26	97.38	72.66	79.18	88.97	99.21	92.33	98.62	4.91	82.91	98.68	79.96	83.09
JGLTM	**80.91**	**98.43**	**74.41**	**80.89**	**90.04**	**99.45**	**92.85**	**99.08**	**4.65**	**83.72**	98.69	**82.35**	**84.04**

**Table 6:** Comparison of results trained on different training sets.

Train Set	Valid Set	AP	PCK
CDEHP	CDEHP	80.91	80.89
CDEHP-E (CDEHP + indoor set)	CDEHP	79.63	79.03

**Table 7:** Action-wise result comparison on CDEHP dataset in terms of the AP metric. Slow actions, medium actions, and fast actions are included in the top, middle, and bottom parts, respectively, while we separate the table into three parts in terms of the action speed. Best in bold, second-best underlined.

Action	Hour- Glass	Simple Baseline	Higher HRNet	LSTM- CPM	DKD	DCPose	Token- Pose	FAMI- Pose	VitPose	tDense- RNN	JGLTM- w/o-Global	JGLTM
walking	68.99	77.63	73.40	42.11	80.64	77.50	78.06	81.37	77.40	80.52	79.41	**84.10**
picking up	73.39	73.94	72.14	56.86	76.66	73.58	75.20	75.42	**79.02**	75.01	75.22	75.87
crawling	62.79	64.39	62.65	31.27	66.79	65.04	68.38	68.37	**71.52**	70.02	68.96	71.48
sweeping	73.94	75.99	73.08	61.92	76.35	73.87	74.33	78.93	**78.95**	78.23	78.30	76.42
shuttlecock kicking	86.25	84.26	84.88	71.64	86.69	84.62	87.13	88.08	87.92	87.54	87.12	**88.75**
Average	73.07	75.24	73.23	52.76	77.43	74.92	76.62	78.43	78.94	78.27	77.80	**79.32**
squat jump	89.42	89.35	88.71	78.49	89.75	87.80	88.81	89.06	87.87	89.93	88.84	**90.02**
frog jump	79.99	79.11	79.45	57.50	82.63	80.89	82.51	**84.66**	81.85	83.09	83.60	83.74
boxing	81.50	80.64	75.25	71.45	80.66	81.31	81.82	82.09	81.49	**83.58**	82.90	81.90
cartwheel	57.49	58.06	56.80	36.67	60.20	59.48	60.59	**68.91**	60.70	63.37	59.66	64.48
rope skipping	75.30	75.18	73.10	65.75	76.76	77.62	77.98	78.95	77.33	78.18	78.25	**79.08**
sit-up jump	74.94	74.75	73.02	59.31	75.46	73.22	74.40	76.34	76.30	**77.86**	74.65	76.40
kicking	69.45	72.45	72.48	60.81	75.99	73.85	76.95	71.58	75.40	77.33	**79.54**	78.79
jump shot	74.30	75.68	72.59	58.02	77.90	76.41	77.10	79.68	**81.34**	79.52	79.32	80.08
spinning	69.11	**75.39**	72.27	56.00	74.37	73.54	71.85	74.91	73.38	74.26	73.10	74.61
throwing	74.20	75.08	70.94	57.46	74.49	75.29	73.93	77.56	76.95	**78.04**	77.46	76.66
Average	74.57	75.57	73.46	60.15	76.82	75.94	76.59	78.37	77.26	78.52	77.73	**78.58**
jumping jack	**96.30**	96.02	95.54	81.88	95.88	95.00	95.62	96.02	95.34	95.94	95.00	95.25
running	73.90	76.91	73.60	54.61	79.50	78.92	79.73	77.24	81.62	80.60	82.30	**83.50**
burpee	72.81	73.62	71.45	46.70	75.38	74.26	75.94	76.61	**79.19**	77.60	75.87	77.36
mopping	69.19	73.86	71.45	49.79	74.05	74.26	70.92	**77.21**	76.48	76.37	72.81	75.56
cycling	72.31	77.69	77.12	66.37	79.93	75.95	80.55	77.60	77.58	81.03	82.58	**83.81**
big jump	92.11	92.18	92.66	77.56	93.01	91.76	**94.37**	94.25	93.48	93.16	93.74	94.09
long jump	69.71	70.13	69.17	54.03	71.39	72.23	72.15	**75.05**	73.27	73.52	74.32	74.81
crotch high five	87.89	88.68	87.79	74.58	89.07	89.80	**90.66**	89.49	88.66	90.32	90.53	90.63
alternate jumping lunge	77.88	80.24	77.28	66.34	80.60	79.94	80.54	78.86	80.83	81.68	80.76	**82.85**
jump fwd/ bwd/left/right	87.56	**87.87**	86.33	80.19	87.84	87.58	86.81	86.89	86.32	87.37	86.16	86.94
Average	79.97	81.72	80.11	65.20	82.66	81.82	82.73	82.92	83.28	83.76	83.41	**84.48**

## Data Availability

The datasets used and analysed during the current study are available online. All data generated or analysed during this study are included in this article.

## References

[1] Xiao B., Wu H., Wei Y. Simple baselines for human pose estimation and tracking. Proceedings of the European Conference on Computer Vision (ECCV).

[2] Sun K., Xiao B., Liu D., Wang J. Deep high-resolution representation learning for human pose estimation. Proceedings of the IEEE/CVF Conference on Computer Vision and Pattern Recognition.

[3] Li Y., Zhang S., Wang Z., Yang S., Yang W., Xia S.T., Zhou E. Tokenpose: Learning keypoint tokens for human pose estimation. Proceedings of the IEEE/CVF International Conference on Computer Vision.

[4] Ma H., Wang Z., Chen Y., Kong D., Chen L., Liu X., Yan X., Tang H., Xie X. (2022). Ppt: Token-pruned pose transformer for monocular and multi-view human pose estimation. Proceedings of the European Conference on Computer Vision.

[5] Luo Y., Ren J., Wang Z., Sun W., Pan J., Liu J., Pang J., Lin L. Lstm pose machines. Proceedings of the IEEE Conference on Computer Vision and Pattern Recognition.

[6] Liu Z., Chen H., Feng R., Wu S., Ji S., Yang B., Wang X. Deep dual consecutive network for human pose estimation. Proceedings of the IEEE/CVF Conference on Computer Vision and Pattern Recognition.

[7] Liu Z., Feng R., Chen H., Wu S., Gao Y., Gao Y., Wang X. Temporal feature alignment and mutual information maximization for video-based human pose estimation. Proceedings of the IEEE/CVF Conference on Computer Vision and Pattern Recognition.

[8] Gai D., Feng R., Min W., Yang X., Su P., Wang Q., Han Q. (2023). Spatiotemporal learning transformer for video-based human pose estimation. IEEE Trans. Circuits Syst. Video Technol..

[9] Pfister T., Charles J., Zisserman A. Flowing convnets for human pose estimation in videos. Proceedings of the IEEE International Conference on Computer Vision.

[10] Song J., Wang L., Van Gool L., Hilliges O. Thin-slicing network: A deep structured model for pose estimation in videos. Proceedings of the IEEE Conference on Computer Vision and Pattern Recognition.

[11] Wei S.E., Ramakrishna V., Kanade T., Sheikh Y. Convolutional pose machines. Proceedings of the IEEE Conference on Computer Vision and Pattern Recognition.

[12] Girdhar R., Gkioxari G., Torresani L., Paluri M., Tran D. Detect-and-track: Efficient pose estimation in videos. Proceedings of the IEEE Conference on Computer Vision and Pattern Recognition.

[13] He K., Gkioxari G., Dollár P., Girshick R. Mask r-cnn. Proceedings of the IEEE International Conference on Computer Vision.

[14] Wang M., Tighe J., Modolo D. Combining detection and tracking for human pose estimation in videos. Proceedings of the IEEE/CVF Conference on Computer Vision and Pattern Recognition.

[15] Feng R., Gao Y., Tse T.H.E., Ma X., Chang H.J. Diffpose: Spatiotemporal diffusion model for video-based human pose estimation. Proceedings of the IEEE/CVF International Conference on Computer Vision.

[16] Calabrese E., Taverni G., Awai Easthope C., Skriabine S., Corradi F., Longinotti L., Eng K., Delbruck T. DHP19: Dynamic vision sensor 3D human pose dataset. Proceedings of the IEEE/CVF Conference on Computer Vision and Pattern Recognition Workshops.

[17] Xu L., Xu W., Golyanik V., Habermann M., Fang L., Theobalt C. Eventcap: Monocular 3d capture of high-speed human motions using an event camera. Proceedings of the IEEE/CVF Conference on Computer Vision and Pattern Recognition.

[18] Scarpellini G., Morerio P., Del Bue A. Lifting monocular events to 3d human poses. Proceedings of the IEEE/CVF Conference on Computer Vision and Pattern Recognition.

[19] Zou S., Guo C., Zuo X., Wang S., Wang P., Hu X., Chen S., Gong M., Cheng L. Eventhpe: Event-based 3d human pose and shape estimation. Proceedings of the IEEE/CVF International Conference on Computer Vision.

[20] Goyal G., Di Pietro F., Carissimi N., Glover A., Bartolozzi C. Moveenet: Online high-frequency human pose estimation with an event camera. Proceedings of the IEEE/CVF Conference on Computer Vision and Pattern Recognition.

[21] Shao Z., Wang X., Zhou W., Wang W., Yang J., Li Y. (2024). A temporal densely connected recurrent network for event-based human pose estimation. Pattern Recognit..

[22] Vaswani A., Shazeer N., Parmar N., Uszkoreit J., Jones L., Gomez A.N., Kaiser Ł., Polosukhin I. (2017). Attention is all you need. Adv. Neural Inf. Process. Syst..

[23] Dosovitskiy A., Beyer L., Kolesnikov A., Weissenborn D., Zhai X., Unterthiner T., Dehghani M., Minderer M., Heigold G., Gelly S. (2020). An image is worth 16x16 words: Transformers for image recognition at scale. arXiv.

[24] Yang S., Quan Z., Nie M., Yang W. Transpose: Keypoint localization via transformer. Proceedings of the IEEE/CVF International Conference on Computer Vision.

[25] Yuan Y., Fu R., Huang L., Lin W., Zhang C., Chen X., Wang J. (2021). Hrformer: High-resolution vision transformer for dense predict. Adv. Neural Inf. Process. Syst..

[26] Mao W., Ge Y., Shen C., Tian Z., Wang X., Wang Z. (2021). Tfpose: Direct human pose estimation with transformers. arXiv.

[27] Li H., Shi B., Dai W., Zheng H., Wang B., Sun Y., Guo M., Li C., Zou J., Xiong H. Pose-oriented transformer with uncertainty-guided refinement for 2d-to-3d human pose estimation. Proceedings of the AAAI Conference on Artificial Intelligence.

[28] Sabater A., Montesano L., Murillo A.C. Event transformer. a sparse-aware solution for efficient event data processing. Proceedings of the IEEE/CVF Conference on Computer Vision and Pattern Recognition.

[29] Zhang J., Dong B., Zhang H., Ding J., Heide F., Yin B., Yang X. Spiking transformers for event-based single object tracking. Proceedings of the IEEE/CVF Conference on Computer Vision and Pattern Recognition.

[30] Cheng B., Xiao B., Wang J., Shi H., Huang T.S., Zhang L. Higherhrnet: Scale-aware representation learning for bottom-up human pose estimation. Proceedings of the IEEE/CVF Conference on Computer Vision and Pattern Recognition.

[31] Hua G., Li L., Liu S. (2020). Multipath affinage stacked—Hourglass networks for human pose estimation. Front. Comput. Sci..

[32] Nie X., Li Y., Luo L., Zhang N., Feng J. Dynamic kernel distillation for efficient pose estimation in videos. Proceedings of the IEEE/CVF International Conference on Computer Vision.

[33] Xu Y., Zhang J., Zhang Q., Tao D. (2023). Vitpose++: Vision transformer for generic body pose estimation. IEEE Trans. Pattern Anal. Mach. Intell..

